# Lysis Profiles of *Salmonella* Phages on *Salmonella* Isolates from Various Sources and Efficiency of a Phage Cocktail against *S.* Enteritidis and *S.* Typhimurium

**DOI:** 10.3390/microorganisms7040100

**Published:** 2019-04-05

**Authors:** Kantiya Petsong, Soottawat Benjakul, Soraya Chaturongakul, Andrea I. Moreno Switt, Kitiya Vongkamjan

**Affiliations:** 1 Department of Food Technology, Prince of Songkla University, Hat Yai 90112, Thailand; ann-kantiya@hotmail.com (K.P.); Soottawat.b@psu.ac.th (S.B.); 2 Department of Microbiology, Mahidol University, Ratchathewi, Bangkok 10400, Thailand; soraya.cha@mahidol.ac.th; 3 Escuela Medicina Veterinaria, Facultad de Ciencias de la Vida, Universidad Andres Bello, Republica 440, 8370251 Santiago, Chile; andrea.moreno@unab.cl

**Keywords:** *Salmonella* phage, animal farm, phage host range, phage cocktail, *Salmonella* serovar, chicken meat, sunflower sprout

## Abstract

*Salmonella enterica* serovar Enteritidis and *Salmonella enterica* serovar Typhimurium are major foodborne pathogens of concern worldwide. Bacteriophage applications have gained more interest for biocontrol in foods. This study isolated 36 *Salmonella* phages from several animal farms in Thailand and tested them on 47 *Salmonella* strains from several sources, including farms, seafood processing plant and humans in Thailand and USA. Phages were classified into three major groups. The estimated phage genome size showed the range from 50 ± 2 to 200 ± 2 kb. An effective phage cocktail consisting of three phages was developed. Approximately 4 log CFU/mL of *S.* Enteritidis and *S.* Typhimurium could be reduced. These phages revealed a burst size of up to 97.7 on *S.* Enteritidis and 173.7 PFU/cell on *S.* Typhimurium. Our phage cocktail could decrease *S.* Enteritidis on chicken meat and sunflower sprouts by 0.66 log CFU/cm^2^ and 1.27 log CFU/g, respectively. *S.* Typhimurium on chicken meat and sunflower sprouts were decreased by 1.73 log CFU/cm^2^ and 1.17 log CFU/g, respectively. Overall, animal farms in Thailand provided high abundance and diversity of *Salmonella* phages with the lysis ability on *Salmonella* hosts from various environments and continents. A developed phage cocktail suggests a potential biocontrol against *Salmonella* in fresh foods.

## 1. Introduction

*Salmonella enterica* serovars Enteritidis and Typhimurium have been reported as the most common causes of salmonellosis outbreaks related to food contamination [1]. The US Centers for Disease Control and Prevention (CDC) reported an estimation of 1 million domestically acquired salmonellosis cases with 19,000 hospitalizations and 380 deaths [2]. Animal farms are typically associated with the presence of diverse *Salmonella* serovars [3,4]. The environments around farms such as feedstuff, soil, water, and feces are common sources of *Salmonella* [4]. Distribution of *Salmonella* on farms leads to the likelihood of *Salmonella* contamination in various food of animal origin and produce. *Salmonella* serovars predominantly present in food can be differed, for example, serovars Enteritidis and Typhimurium are common in eggs [5], serovars Kentucky and Enteritidis are common in poultry [6], serovars Newport, Infantis and Javiana are common in fruits and vegetables [7]. The environments of food processing plant have been linked to occurrence of diverse *Salmonella* serovars. For example, serovars Weltevreden [8,9], Anatum, Enteritidis, Newport, and Typhimurium have been previously detected in wastewater from the plant [10]. *Salmonella* serovars predominant in human are also of crucial concern. These include *Salmonella* serovars Weltevreden, Enteritidis and Anatum which have been reported as the most common serovars isolated from human in Thailand [11]. The occurrence of *Salmonella* contaminated in poultry meat has been often reported [12]. Fresh produce such as sprout has also been reported as the high risk food that is commonly been contaminated by *Salmonella* [12,13]. Control of *Salmonella* in both food categories is thus crucial.

Bacteriophages (phages) are viruses of bacteria which are adapted to be very specific for infecting and killing bacterial hosts [14]. The habitat of phages is normally related to their hosts as predator and prey [15]. Phage application as a biocontrol agent against pathogens has been reported the outstanding properties over antibiotics (e.g., specificity to target hosts) [14] or chemical agents which are commonly used in meat products and may have some negative effects on human health [16]. Certain phages have been shown to have minimal effects on the change of quality and sensory properties of specific foods [17]. To improve the antibacterial efficiency and avoid phage-resistant bacteria from using phage-based biocontrol, phage cocktail is an alternative approach. Several studies have reported the success in using effective phage cocktails to control *Salmonella* serovars in various food products, including raw meat, fresh produce and ready-to-eat foods [18,19,20].

Phage abundance and diversity recovered from particular sources can provide a better understanding on the phage-host relationship. Phage lysis profiles obtained from testing phages against *Salmonella* strains from various sources are useful information for further development of phage-based control for targeting *Salmonella* serovars predominant in different sources. This study aimed to isolate *Salmonella* phages from animal farms (poultry, swine, goat and bovine) in Thailand and investigate phage lysis profiles on *Salmonella* strains from at least two geographical regions (Thailand and USA) isolated from different sources, including animal farms, food processing plants and humans that have a history of *Salmonella* infection. Phages presenting the highest lysis ability against the two most common *Salmonella* serovars (Enteritidis and Typhimurium) were further developed as a phage cocktail. One-step growth curves of all phages included in the cocktail were studied to determine phage biology including latent period and burst size. Our developed phage cocktail was evaluated the effectiveness in reducing *S.* Enteritidis and *S*. Typhimurium in vitro and in fresh foods (raw chicken meat and sunflower sprouts) during storage at 4 °C.

## 2. Materials and Methods

### 2.1. Sample Collection

A total of 12 samples were collected from six animal farms, including poultry, swine, goat, and bovine farms in Songkhla province, Thailand between January 2014 and October 2016. Of six farms, three of which were commercial farms (poultry, swine and goat) and three others were free range farms (poultry and bovine). Approximately 500 g of each randomly selected sample (feces, feed, soil and drinking water) was collected using a sterile spatula and transferred to a sterile bag. Feces samples were collected from the floor inside the pens of the commercial farms where animals resided. For free range farms, feces samples were collected from random open areas where animals resided. Feed samples were taken from the storage containers freshly prepared for feeding animals. Soil samples were taken from the free range farms only and from random open areas where animals resided. Drinking water in farms was also collected. Each sample was kept in a cooler box containing ice packs and transferred to a laboratory. Collected samples were stored at 4 °C until analysis in the laboratory.

### 2.2. Bacterial Strains Used in the Study

*Salmonella* strains used in this study consisted of 47 *Salmonella* strains presenting 28 common serovars (Table 1). All *Salmonella* strains tested were the representative of predominant serovars isolated from various sources, including animal farms, food processing plants and humans in Thailand and USA. A total of 23 strains of *Salmonella* isolated from animal farms and human in the USA were obtained from Food Safety Laboratory, Cornell University (indicated with a prefix of “FSL”). Other 24 strains of *Salmonella* were previously isolated from animal farms, food processing plants and humans in Thailand. These were obtained from the Faculty of Microbiology, Mahidol University and the Faculty of Agro-Industry, Prince of Songkla University, Thailand (indicated with a prefix of “PSU-BS-”). *Salmonella* strains were kept in 15% glycerol at −80 °C as working stocks. For overnight culture, an isolated colony of *Salmonella* from Tryptone Soya Agar (TSA; Oxoid, Hampshire, UK) was transferred in 5 mL of Tryptone Soya Broth (TSB; Oxoid, Hampshire, UK) and incubated at 37 °C for 16–18 h.

### 2.3. Isolation and Purification of Salmonella Phages

*Salmonella* phages were isolated using enrichment isolation with a multi-strain *Salmonella* cocktail whose serovars are shown to be predominant in Thai animal farms (Agona H2-016, Anatum A4-525, Give H2-018, Kentucky W1-010, Stanley H2-002, Typhimurium H2-001, and Virchow H2-117) [11,21]. Briefly, 25 g of each sample was enriched with 225 mL of TSB and 2.5 mL of host mixture. After filtration of the mixture through 0.45 µm and 0.22 µm syringe filters, filtrate (100 µL) was used to prepare the double layer by mixing with each host strain previously grown in TSB for 16–18 h at 37 °C. For each overlay, 300 µL of the 1:10 dilution of the overnight host strain was mixed with 4 mL of 0.7% TSA, followed by incubation at 37 °C for 18–24 h. Plaques were observed on each host lawn. A distinct isolated plaque was selected and suspended in 300 µL of Phosphate Buffered Saline (PBS, (pH 7.4), 137 mM NaCl, 2.7 mM KCl, 4.3 mM Na_2_HPO_4_, 1.4 mM KH_2_PO_4_) for purification. Serial dilutions were performed and appropriate dilution was subjected to three passages with a specific host that showed a positive result, using a double layer agar technique [4].

### 2.4. Lysate Preparation and Titer Determination of Salmonella Phages

An isolated plaque from the third purification passage was used to prepare 10-fold serial dilutions in PBS. Appropriate dilutions were used to prepare the overlay with a given host to yield semi-confluent lysis. Overlay was harvested with 10 mL of Salt-Magnesium buffer (SM buffer), followed by centrifugation at 3213× *g* for 15 min at 4 °C. Supernatant was filtered through a 0.22 µm syringe filter and phage lysates were kept at 4 °C. Each phage lysate was serially diluted in PBS and 100 µL of each dilution was mixed with 300 µL of host, then the mixture was poured on bottom agar [22]. Phage titers were determined after incubation at 25 °C for 16–18 h by counting plaques present on each plate of a given dilution [22].

### 2.5. Determination of Lysis Profiles of Salmonella Phages

Lysis profile for each phage was determined by a spot test on bacterial lawn of a given *Salmonella* strain in the collection included in this study. Briefly, 5 µL of each phage lysate representing 10^8^ PFU/mL were spotted on the bacterial host lawn prepared as mentioned above but without filtrate. Phage lysis patterns were determined after 18–24 h of incubation at 25 °C. The experiment was performed in independent triplicates. Phage lysis patterns were analyzed by converting a positive lysis (zone of lysis on a spot) to a score of 1 and negative results were converted to a score of 0. A heatmap representing lysis groups was generated by cluster analysis, following Vongkamjan et al. [22] with Ward’s method of binary distance, using the R software program (R development Core Team 2012 [23].

### 2.6. Genome Size Determination of Salmonella Phages

Representative *Salmonella* phages from each sample source were selected for genome size determination. A total of 17 phage isolates were included for Pulsed-Field Gel Electrophoresis (PFGE) analysis. Agarose plugs were prepared by mixing equal volume (55 µL) of a given phage lysate with high titer of approximately 10^6^–10^8^ PFU/mL and 1.3% low melting point agarose. Plugs were kept at low temperature (4 °C) in order to solidify for 1 h. Plugs were loaded into 1% agarose gel and electrophoresis was performed in 0.5X TBE buffer using CHEF-DR III system (Bio-Rad, Hercules, CA, USA). PFGE was performed for 20 h with 0.5–5 s of switch time. Two size markers were included; CHEF DNA size standard of 8–48 kb ladder and CHEF DNA size standard lambda λ ladder 0.05–1 Mb (both from Bio-Rad, Hercules, CA, USA) [4,22].

### 2.7. Development of a Phage Cocktail Targeting Two Major Salmonella Serovars

To develop a *Salmonella* phage cocktail against *S.* Enteritidis and *S.* Typhimurium, nine phages which showed the strong lysis ability on *S.* Enteritidis and *S.* Typhimurium (KP1, KP2, KP4, KP5, KP9, KP34, KP36, KP49, and KP50) were ordered their lysis ability by a spotting test and efficiency of plating (EOP) using method modified from previous study [24]. In this study, *S.* Enteritidis FSL S5-371 and *S.* Typhimurium H2-001 were used as the target hosts. *S.* Anatum FSL A4-525 was used as the reference host of phages KP1, KP2, KP5 and KP9. *S.* Virchow H2-117 was used as the reference host of phages KP34 and KP36. *S.* Agona H2-016 was used as the reference host of phages KP49 and KP50. Three *Salmonella* phages which showed the highest lysis ability were selected to prepare a cocktail using a ratio of 1:1:1 for each phage.

#### 2.7.1. Spotting Assay

Each *Salmonella* phage dilutions ranging in concentration from 10^3^–10^7^ PFU/mL were spotted on bacterial lawn of *S.* Enteritidis or *S.* Typhimurium prepared as mentioned above. Immediately after spotting, the plates were incubated at 25 °C for 16–18 h. The clear zone or visible plaques formed by serial dilutions on the plate were determined as +++, confluent lysis (clear spot); ++, semi-confluent lysis (semi-clear); +, turbidity without plaque formation. The experiment was repeated three times for each phage.

#### 2.7.2. Efficiency of Plating (EOP) Assay

Similar to a spotting assay, each *Salmonella* phage dilution ranging in concentrations from 10^3^–10^7^ PFU/mL were spotted on bacterial lawn of *S.* Enteritidis or *S.* Typhimurium. EOP assay replicates for a particular phage were done in parallel on both reference and target hosts. The EOP was calculated by the ratio of the average PFU on a target host to the average PFU on a corresponding reference host. EOP values were presented in 3 levels; high production (EOP ≥ 0.5), medium production (0.01 ≤ EOP < 0.5) and low production (0.0001 < EOP < 0.01).

### 2.8. One-Step Growth Curve

A one-step growth curve for three phages included in the phage cocktail were investigated following a protocol of Bao et al. [25] with modifications. The bacterial hosts *S.* Enteritidis and *S.* Typhimurium grown overnight (10^7^ CFU/mL) in TSB as mentioned above were mixed with 10^8^ PFU/mL and 10^9^ PFU/mL of phage to a final volume of 30 mL to represent the multiplicity of infection (MOI) of 10 and 100, respectively. The co-culture was incubated at 37 °C (220 rpm) for an initial attachment for 20 min, followed by centrifugation of the sample at 6000× *g* for 10 min at 4 °C to remove the excess phage as the supernatant. Cell pellets were re-suspended with the same volume (30 mL) as pre-centrifugation with TSB and resumed to incubation for additional 60 min. Lysate (1 mL) was taken every 5 min for the standard plaque count assay (in triplicates) to determine the number of phages obtained from each period. Latent period was defined as the time interval between the adsorption (not including 20 min of pre-incubation) and the beginning of the first burst, as indicated by the initial rise in the phage titer. Burst size was calculated as the ratio of the final count of liberated phage particles to the initial count of infected bacterial cells during the latent period.

### 2.9. Efficiency of Phage Cocktail to Reduce S. Enteritidis and S. Typhimurium In Vitro and Evaluation of Phage-Resistance in Salmonella after Treated with Phage Cocktail

An overnight culture of *S.* Enteritidis and *S.* Typhimurium prepared as mentioned above (10 mL) was separately centrifuged at 6000× *g* for 10 min at 4 °C. To wash cell pellets, 5 mL of PBS were added and centrifuged at the same conditions three times. Washed *Salmonella* pellets were suspended in TSB and diluted to approximately 10^5^ CFU/mL. Phage cocktail stock was diluted with TSB to achieve phage concentration at 10^7^ PFU/mL. Each *Salmonella* strain suspension and phage cocktail preparation were mixed at a ratio of 1:1 by volume and incubated at 37 °C in a shaking incubator (ThermoStable^TM^ IS-30 model, DAIHAN Scientific, Gangwon-do, Korea) at 220 rpm for 12 h. Controls included in the study were only *S.* Enteritidis and *S.* Typhimurium cultured in TSB. The cell numbers of *S.* Enteritidis and *S.* Typhimurium from the treatments and controls at each temperature tested were enumerated every 4 h interval by a spread plate on TSA. After 12 h of experiment, samples were collected for the analyses of phage resistance.

The changes in resistance phenotype of *S.* Enteritidis and *S.* Typhimurium after treated with a phage cocktail were evaluated by a spotting test. Five colonies of *S.* Enteritidis and *S.* Typhimurium from controls and treatments of phage cocktail recovered from TSA after 12 h of experiment above were re-cultured in TSB, followed by incubation at 37 °C for 16–18 h. Each culture was used to prepare an overlay for a spotting test. Serial dilutions (10^3^–10^7^ PFU/mL) of phage cocktail and each individual phage mixed in a cocktail were spotted on each bacterial lawn. Phage lysis patterns were determined after 18–24 h of incubation at 25 °C.

### 2.10. Efficiency of Phage Cocktail to Reduce S. Enteritidis and S. Typhimurium in Foods

Chicken meat and sunflower sprouts were selected as representative foods which commonly have *Salmonella* contamination. Samples were purchased from the supermarkets in Hat Yai, Thailand. To eliminate *Salmonella* that may be present, the samples were soaked in 50 ppm free chlorine concentration solution for 5 min [26]. Subsequently, the samples were soaked and washed in sterile distilled water for 5 min for 3 times. Chicken breast was aseptically cut into pieces of approximately 5 × 5 cm^2^ and sunflower sprout was aseptically weight approximately 5 g. Overnight culture of *S.* Enteritidis and *S.* Typhimurium was prepared. Cell pellets were suspended with PBS and diluted to obtain the final concentration of 10^5^ CFU/mL. One milliliter of each *Salmonella* strain was evenly spiked to the surface of each piece of chicken breast and sunflower sprout in a sterile Whirl-Pak bag to achieve the inoculation level of approximately 10^5^ CFU/piece or 10^5^ CFU/5 g. Samples were left on clean bench for 10 min to allow the cells to adapt to the conditions on food samples tested. A phage cocktail preparation (1 mL containing 10^7^ PFU/mL) was evenly spiked to each piece of chicken and sunflower sprout in a sterile Whirl-Pak bag. PBS buffer (1 mL) was added to sample inoculated with *Salmonella* as control. Treatments and control were stored at 4 °C. Number of *Salmonella* cells was enumerated on day 0, 1, 2, 3, and 4 on Xylose-Lysine-Desoxycholate agar (XLD; Oxoid, Hampshire, UK).

### 2.11. Statistical Analysis

Differences of the bacterial counts between control and treatment by a phage cocktail in this study were analyzed by Student’s *t*-test for both in vitro and in food models. The Analysis of variance (ANOVA) was used to compare differences between the storage period for a given strain. Comparison of means was carried out by Duncan’s multiple range tests. Significance was declared at *p* < 0.05 using the statistical Package for Social Science (SPSS 10.0 for windows, SPSS Inc., Chicago, IL, USA).

## 3. Results

### 3.1. Recovery of Salmonella Phages from Various Animal Farms in Thailand

Of 12 samples collected from three sampling visits to various animal farm environments in Songkhla province, Thailand, 36 *Salmonella* phages were obtained (Table 2). Three samples from sampling visit 1 from commercial farms represented two phages. Vast numbers of phages (34 phages) were obtained from the other 9 samples from two samplings at free range farms. Genome sizes of representative phages from different animal farms and different sources were estimated by PFGE analysis. Of the 17 representative phages, isolated phages from feces, feed, soil and drinking water from poultry farms, they showed an estimated genome size in the range of 50 ± 2 kb to 105 ± 2 kb (Table 3). An estimated genome size of 200 ± 2 kb was observed in phages isolated from goat feces. Whereas, phages isolated from bovine feces showed an estimated genome size of 50 ± 2 kb and 60 ± 2 kb. Overall, phages isolated in this study showed an estimated genome size in the range of 50 ± 2 kb to 200 ± 2 kb.

### 3.2. Lysis Profiles of Salmonella Phages from Animal Farms on Salmonella Strains from Various Sources in Thailand and USA 

**Figure 1:**
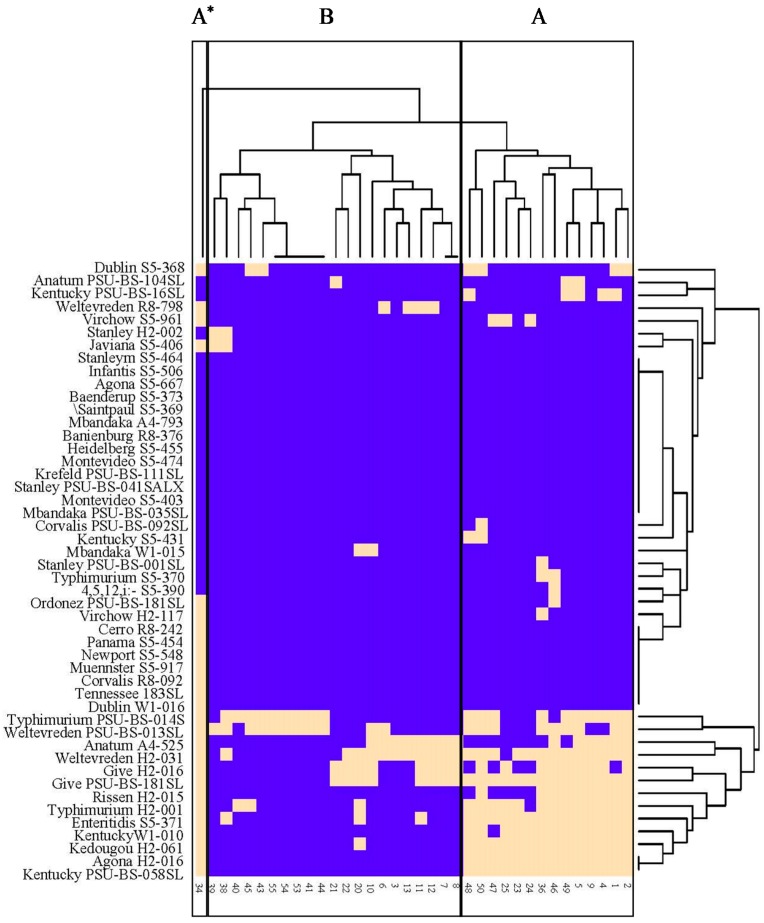
Heatmap representation of lysis profiles of 36 *Salmonella* phages tested (vertical axis) on 47 *Salmonella* host strains (horizontal axis) from Thailand and USA Phages were classified into three groups, including (**A**) broad, (**B**) narrow and (A*) special broad host range. Beige areas indicate lysis and darker areas indicate no lysis.

All 36 *Salmonella* phages obtained in this study were tested on 47 *Salmonella* strains representing 28 serovars. These phages were classified into three groups based on the host range, including (A) broad, (B) narrow and (A*) special broad host range (Figure 1). In group A, 14 phages showed strong lysis ability with *Salmonella* strains from Thailand but lower weaker lysis ability with *Salmonella* strains from USA However, several phages showed ability to lyse *Salmonella* strains from both continents and different sources. In group B, 21 phages showed the ability to lyse *Salmonella* strains mostly isolated from Thailand, especially *Salmonella* isolated from animal farms. One unique phage (KP34) isolated from a commercial poultry farm was classified into the special broad host range group. This phage showed the broadest host range among isolated phages with the ability to lyse over 60% of *Salmonella* strains from Thailand and 48% of *Salmonella* strains from USA Overall, phages isolated in this study presented different spectrum to lyse host strains from both continents and different sources.

### 3.3. Phage Susceptibility of Different Salmonella Serovars from Various Sources

All 36 *Salmonella* phages obtained in this study were tested on 47 *Salmonella* strains of different major serovars previously isolated from the environments related to animal farms, animal slaughterhouse and food processing plant and from humans. High susceptibility to phage lysis was observed among *Salmonella* strains mostly isolated from animal farms and slaughterhouse in Thailand (Table 4), including serovars Agona, Give, Kedougou, Kentucky, Typhimurium and Weltevreden. Two additional strains showing high susceptibility to phage lysis included serovars Anatum and Enteritidis. Other five serovars, including Kentucky, Rissen, Dublin, Virchow and Weltevreden from diverse sources from both Thailand and USA presented medium susceptibility to phage lysis. Several serovars showed low susceptibility to phage lysis. However, among serovars classified in this group, most *Salmonella* strains were isolated from USA These included serovars Agona, Braenderup, Heidelberg, Infantis, Javiana, Kentucky, Mbandaka, Montevideo, Muenster, Newport, Oranienburg, Panama, Saintpaul, Stanley and Typhimurium.

### 3.4. Development of a Phage Cocktail

**Figure 2:**
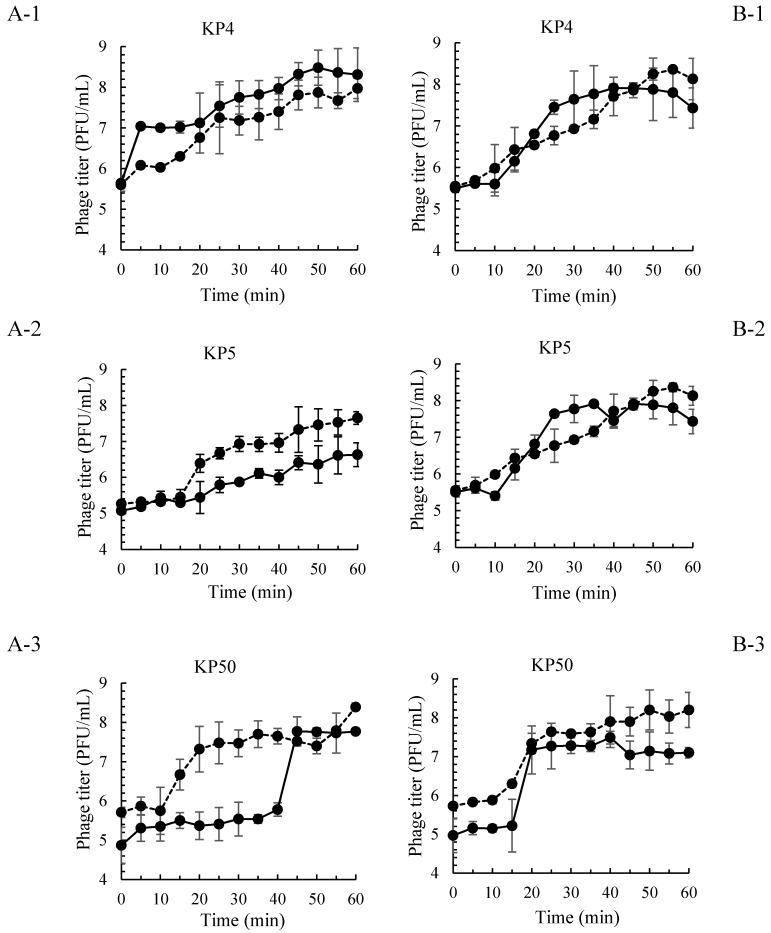
One-step growth curve of phage KP4, KP5 and KP50 on *S.* Enteritidis (**A-1**, **A-2** and **A-3**) and *S.* Typhimurium (**B-1**, **B-2** and **B-3**). MOI 100 presented by 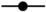 and MOI 10 presented by 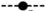. Bars represent the mean standard deviation (*n* = 3).

Of 36 isolated *Salmonella* phages evaluated for a lysis profile, 9 phages showed strong lysis ability on *S.* Enteritidis and *S.* Typhimurium. Based on the highest EOP, three phages (KP4, KP5 and KP50) were selected for the development of a phage cocktail (Table 5). Each phage composed in a phage cocktail preparation showed the latent period on *S.* Enteritidis as 5, 15 and 40 min at MOI 100, and 10, 15 and 10 min at MOI 10 for phages KP4, KP5 and KP50, respectively (Table 6, Figure 2). On *S.* Typhimurium, these phages showed the latent period of 10, 10 and 15 min at MOI 100, and 15, 15 and 10 min at MOI 10 for phages KP4, KP5 and KP50, respectively. Phages KP4, KP5 and KP50 included in a phage cocktail preparation showed large burst sizes as 25.1, 30.1 and 97.7 PFU/cell, respectively on *S.* Enteritidis at MOI 100. At MOI 10, the burst sizes of phages KP4, KP5 and KP50 on *S.* Enteritidis were observed as 16.6, 6.6 and 37.1 PFU/cell, respectively. On the *S.* Typhimurium host, the burst size of phages KP4, KP5 and KP50 was observed as 70.8, 173.7 and 112.2 PFU/cell, respectively at MOI 100, and 19.1, 19.1 and 28.8 PFU/cell, respectively at MOI 10.

### 3.5. Efficiency of Salmonella Phage Cocktail in Reducing S. Enteritidis and S. Typhimurium In Vitro and Different Fresh Foods, and Evidence of Phage-Resistant Development in Salmonella

**Figure 3:**
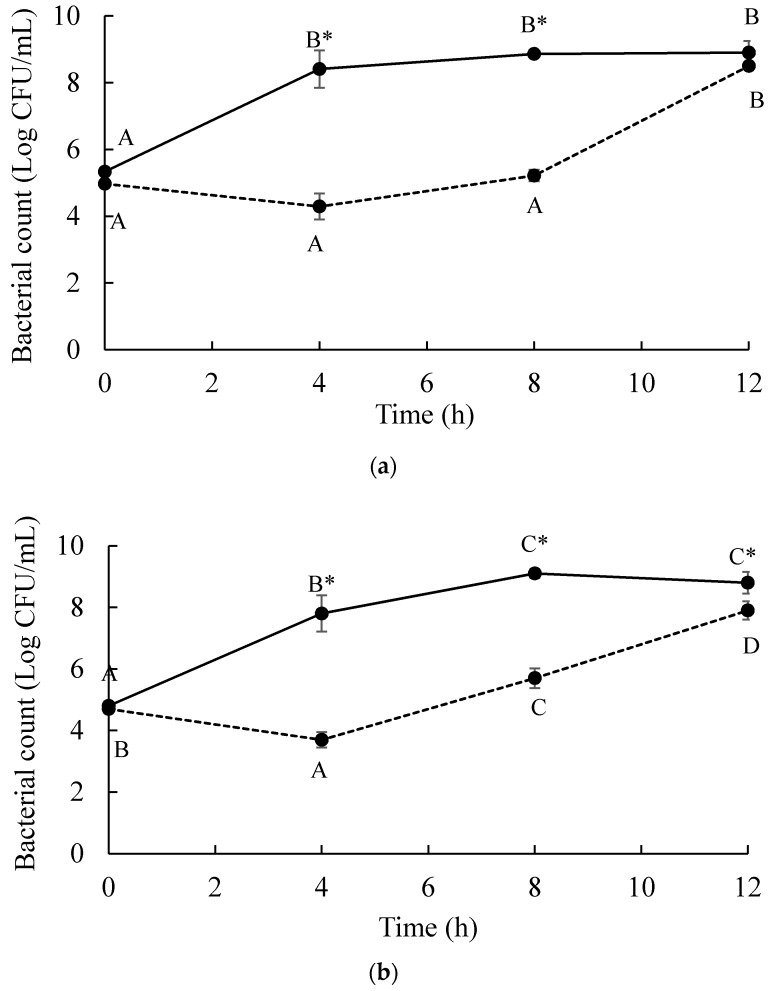
Survival of (**a**) *S.* Enteritidis and (**b**) *S.* Typhimurium treated with a phage cocktail at 37 °C for 12 h. Control (non-phage cocktail treated) presented by 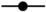 and treatment of a phage cocktail presented by 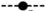. Bars represent the mean standard deviation (*n* = 3). The sign (*) on the lines indicates significant differences (*p* < 0.05) of bacterial count between control and treatment during storage time for a given *Salmonella* strain. Different uppercase letters on the lines indicate significant differences (*p* < 0.05) of bacterial count among days of storage for a given *Salmonella* strain.

**Figure 4:**
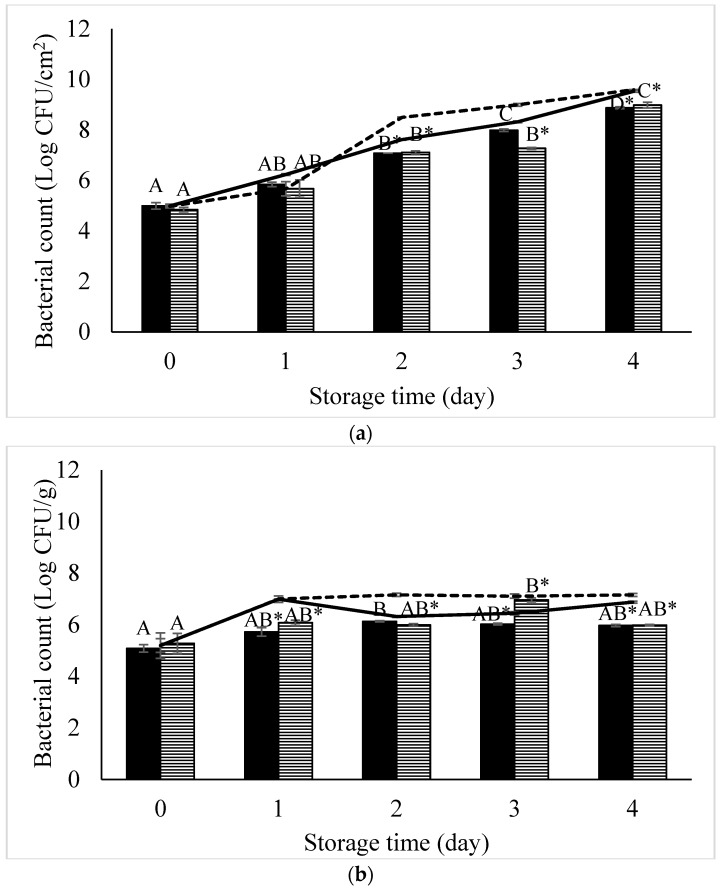
Recovery of *S.* Enteritidis (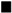) and *S.* Typhimurium (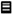) artificially contaminated in (**a**) chicken meat and (**b**) sunflower sprouts treated with a phage cocktail and stored at 4 °C for 4 days. Control (non-phage cocktail treated) of *S.* Enteritidis and S. Typhimurium inoculated on both samples presented by solid line and dashed line, respectively. Bars represent the mean standard deviation (*n* = 3). The sign (*) on the bars indicates significant differences (*p* < 0.05) of bacterial count between control and treatment during storage time for a given *Salmonella* strain. Different uppercase letters on the bars indicate significant differences (*p* < 0.05) of bacterial count among days of storage for a given *Salmonella* strain.

In vitro study showed that a phage cocktail could decrease the numbers of *S.* Enteritidis and *S.* Typhimurium as indicated by the highest reduction of both serovars of more than 4 log CFU/mL after 4 h of a phage cocktail treatment at MOI 100 (Figure 3). During the 8 h treatment, an overall reduction of more than 3 log CFU/mL was observed for both strains tested. In chicken meat artificially contaminated with *Salmonella*, a high reduction of *S.* Enteritidis was observed in phage-treated chicken meat on day 2 (0.53 log CFU/cm^2^) and day 4 (0.66 log CFU/cm^2^) (Figure 4). For *S.* Typhimurium, a high reduction was observed in phage-treated chicken meat on day 2 (1.39 log CFU/cm^2^) and day 3 (1.73 log CFU/cm^2^).

A phage cocktail treatment showed a high reduction of *S.* Enteritidis in sunflower sprouts on day 1 (1.27 log CFU/g) and day 4 (0.91 log CFU/g). For *S.* Typhimurium, a high reduction was observed in phage-treated sunflower sprout on day 2 (1.17 log CFU/g) and day 4 (1.17 log CFU/g). Overall, during 4 days of storage at 4 °C, the numbers of *S.* Enteritidis and *S.* Typhimurium were decreased by 0.66 and 1.73 log CFU/cm^2^ in chicken meat and 1.27 and 1.17 log CFU/g in sunflower sprouts, respectively. Results indicate a potential use of this phage cocktail in chicken meat and sunflower sprouts during a storage condition at 4 °C for 4 days.

Phage-resistant development in *Salmonella* was investigated after a treatment with our phage cocktail preparation. A phage cocktail and individual phages included in phage cocktail preparation were re-tested on sub-cultured *S.* Enteritidis and *S.* Typhimurium recovered from a previous phage challenge study. Sub-cultured *Salmonella* strains previously treated with a phage cocktail presented similar results as the sub-cultured *S.* Enteritidis and *S.* Typhimurium from the non-phage treatment (Table 7). However, individual phages showed similar lysis ability on the sub-cultured *Salmonella* strains from both the previous treatment and the non-phage treatment.

## 4. Discussion

The recovery of high number of *Salmonella* phages in this study suggests that animal farm environments in Songkhla province, Thailand represent an important source of abundant *Salmonella* phages. Typically, *Salmonella* phages have been isolated from diverse animal farms such as poultry farms [25,27], swine farms [28,29], goat farms [30] and bovine farms [4,31]. Our study also suggests that free range farms could yield many phages, especially from soil and feed samples. Similar to previously reported sources of phages, Wongsuntornpoj et al. [31] isolated a number of *Salmonella* phages from small-scale free range cattle farms in Thailand [31]. As the presence of bacteriophage is normally related to bacterial host population [32], samples from free range farms including soil in an open land may have high opportunity to be exposed to various important sources of *Salmonella* such as wild birds, insects, rodent droppings and other carriers [33].

Variations in the genome size of *Salmonella* phages obtained in this study indicate the potential diverse phage groups that may be distributed in farm environments in Thailand. Some identical genome sizes were observed among phages from various farms in different countries (Table 8), suggesting presence of common phage types from these animal farm-related sources. Among *Salmonella* phages isolated from dairy farms in USA, the genome sizes ranging from 22 kb to 156 kb [4], while some phages from dairy farms in Thailand exhibited the genome sizes of 40 kb to 200 kb [31]. Lysis profiles indicated that our isolated phages showed better ability to lyse *Salmonella* strains from animal farms in Thailand as compared to strains from USA. Our findings indicate the relationship between phages and *Salmonella* hosts is due to geological isolation. This phenomenon can be explained by the mechanisms of the phage-host receptors which are related to the evolution as pray-predator [34]. However, the spectrum of phage lysis can be extended across different sources or regions of host habitats. In this study, our isolated phages showed lysis on *Salmonella* strains from diverse sources including environments related to animal farms, animal slaughterhouses, food processing plants and from humans. Phages could also lyse bacterial hosts from different continents. Overall, phages isolated from animal farms in this study could lyse most serovars of *Salmonella* that have been reported as the predominant and prevalent serovars in humans, foods and animal farms in Thailand including *Salmonella* serovars Agona, Anatun, Give, Enteritidis, Kedougou, Kentucky, Typhimurium and Weltevreden [11,21,35,36]. Findings here suggests a potential for phage applications against bacterial hosts from various environments in different food chains or countries.

Phage latent period and burst size are major parameters which play an important role in the host lysis system [42]. Phages which present a short latent period and large burst size will be replicated more quickly and the new phage particles (progeny) could be released more efficiently [25]. Our phages combined in a phage cocktail showed a short latent period and large burst size of up to 97.7 and 173.7 PFU/cell on both *S.* Enteritidis and *S.* Typhimurium, respectively. The range of *Salmonella* phage latent periods has been reported within the range of 15 to 45 min [43]. Abedon et al. [44] reported that shorter phage latent periods could be obtained by higher bacterial densities. It is also suggested that phage exhibiting very short latent periods maybe viewed as specialists for propagation when bacteria within cultures are highly prevalent [44]. Phage burst size has been reported with large variations (5 to 250 PFU/cell) depending on the bacterial strain infected [45]. Overall, the short latent period and large burst size of our phages suggests a rapid replication and effective release of new phage particles from both *S.* Enteritidis and *S.* Typhimurium hosts, thus appropriate for using to control bacterial hosts as reported by previous other studies [46,47].

Our developed phage cocktail showed high efficiency to control *S.* Enteritidis and *S.* Typhimurium in both in vitro study and different food categories that are typically linked to *Salmonella* and foodborne outbreaks. Our phage cocktail could decrease *S.* Enteritidis on chicken meat and sunflower sprouts by 0.66 log CFU/cm^2^ and 1.27 log CFU/g, respectively. *S.* Typhimurium on chicken meat and sunflower sprouts were decreased by 1.73 log CFU/cm^2^ and 1.17 log CFU/g, respectively. In a previous study, Grant et al. [48] demonstrated a < 1 log reduction of *Salmonella* on ground chicken after treatment with the commercial *Salmonella* phage cocktail (Salmonelex^TM^, Micreos Food Safety, Wageningen, The Netherlands) [48]. Another phage cocktail showed a reduction of *S.* Enteritidis and *S.* Typhimurium on chicken breast at 0.9 and 2.2 log CFU/g, respectively within 7 days at 4 °C [20]. Our finding showed no evidence of phage resistance in both *Salmonella* serovars upon phage cocktail treatment. This phenomenon might be related to the biology (latent period and burst size) of the phages included in our phage cocktail. Short periods of time to kill bacterial hosts for phages is an important factor that prevents the occurrence of bacteria resistance to phage [43].

## 5. Conclusions

This study aims to understand the abundance and diversity of *Salmonella* phages in animal farm environments. Phages presenting an ability to lyse *Salmonella* strains from different sources in the food chain and countries were included for development of *Salmonella* phage cocktail. Phages from our collection obtained from animal farms in Thailand indicate diverse and effective phages that can be used as a biocontrol agent to control *S.* Enteritidis and *S.* Typhimurium in different food categories. In addition, phage lysis profiles indicated that our isolated phages showed a high ability to lyse several important serovars of *Salmonella* predominant in various sources and prevalent in different countries. Phages composed in our developed cocktail showed a rapid replication and effective release of new phage particles from both *S.* Enteritidis and *S.* Typhimurium hosts as indicated by short latent period and large burst size. The developed phage cocktail in this study showed to be highly effective for the reduction of *S.* Enteritidis and *S.* Typhimurium in fresh foods, especially those that have been highly related to *Salmonella* contamination and foodborne outbreaks.

## Figures and Tables

**Table 1:** *Salmonella* strains used for phage isolation and determination of phage lysis profiles.

Serovars	Isolates ID	Source (Country)
Agona	H2-016 *	Pig slaughterhouse (Thailand)
	FSL S5-667	Bovine (USA)
Anatum	PSU-BS-104SL	Seafood processing plant (Thailand)
	FSL A4-525 *	Bovine (USA)
Braenderup	FSL S5-373	Human (USA)
Cerro	FSL R8-242	Bovine (USA)
Corvolis	PSU-BS-092SL	Animal farm (Thailand)
	FSL R8-092	Human (USA)
Dublin	W1-016	Dairy farm (Thailand)
	FSL S5-368	Bovine (USA)
Enteritidis	FSL S5-371	Human (USA)
Give	PSU-BS-181SL	Animal farm (Thailand)
	H2-018 *	Dairy farm (Thailand)
Heidelberg	FSL S5-455	Human (USA)
Infantis	FSL S5-506	Human (USA)
Javiana	FSL S5-406	Human (USA)
Kedougou	H2-061	Pig slaughterhouse (Thailand)
Kentucky	W1-010 *	Dairy farm (Thailand)
	PSU-BS-058SL	Chicken farm (Thailand)
	PSU-BS-116SL	Seafood processing plant (Thailand)
	FSL S5-431	Bovine (USA)
Krefeld	PSU-BS-111SL	Seafood processing plant (Thailand)
Mbandaka	W1-015	Dairy farm (Thailand)
	PSU-BS-035SL	Chicken farm (Thailand)
	FSL A4-793	Bovine (USA)
Montevideo	FSL S5-474	Bovine (USA)
	FSL S5-403	Bovine (USA)
Muennster	FSL S5-917	Bovine (USA)
Newport	FSL S5-548	Bovine (USA)
Oranienburg	FSL R8-376	Human (USA)
Ordonez	PSU-BS-181SL	Seafood processing plant (Thailand)
Panama	FSL S5-454	Human (USA)
Rissen	H2-015	Pig slaughterhouse (Thailand)
Saintpaul	FSL S5-369	Human (USA)
Stanley	PSU-BS-001SL	Swine farm (Thailand)
	H2-002 *	Pig slaughterhouse (Thailand)
	PSU-BS-041SALX	Seafood processing plant (Thailand)
	FSL S5-464	Human (USA)
Tennessee	PSU-BS-183SL	Seafood processing plant (Thailand)
Typhimurium	H2-001 *	Pig slaughterhouse (Thailand)
	PSU-BS-014SL	Chicken farm (Thailand)
	FSL S5-370	Human (USA)
Virchow	FSL H2-117 *	Human (Thailand)
	FSL S5-961	Human (USA)
Weltevreden	H2-031	Pig slaughterhouse (Thailand)
	PSU-BS-013SL	Chicken farm (Thailand)
	FSL R8-798	Human (USA)

* indicates strains of *Salmonella* used as hosts for *Salmonella* phage isolation.

**Table 2:** Recovery of *Salmonella* phages from various animal farms in Thailand.

Animal Farms (Sampling Date [mo/yr])	Number of Sample (Number of Phages)
Sampling 1 (04/2014)	
Poultry farm; commercial	1 (1)
Swine farm; commercial	1 (0)
Goat farm; commercial	1 (1)
Sampling 2 (08/2015)	
Bovine farm; free range	1 (3)
Poultry farm; free range	4 (15)
Sampling 3 (10/2016)	
Poultry farm; free range	4 (16)
Total	12 (36)

**Table 3:** Estimated genome size of representative *Salmonella* phages from various animal farms in this study.

*Salmonella* Phage ID	Source	Estimated Genome Size (kb)
KP3	Goat feces	200 ± 2
KP4	Soil (poultry farm 2)	105 ± 2
KP5	Poultry feces (poultry farm 3)	60 ± 2
KP6	Poultry feed (poultry farm 2)	72 ± 2
KP7	Soil (poultry farm 2)	60 ± 2
KP10	Poultry feed (poultry farm 2)	60 ± 2
KP11	Soil (poultry farm 2)	60 ± 2
KP34	Poultry feces (poultry farm 1)	60 ± 2
KP38	Bovine feces	60 ± 2
KP39	Bovine feces	50 ± 2
KP41	Drinking water (poultry farm 3)	97 ± 2
KP43	Poultry feces (poultry farm 3)	60 ± 2
KP49	Soil (poultry farm 3)	60 ± 2
KP50	Poultry feces (poultry farm 3)	103 ± 2
KP53	Poultry feces (poultry farm 3)	50 ± 2
KP54	Soil (poultry farm 3)	50 ± 2
KP55	Poultry feed (poultry farm 3)	60 ± 2

**Table 4:** Phage susceptibility of different *Salmonella* serovars recovered from various sources.

Serovars	Source	% Susceptibility (Level) ^a^	
		Thailand	USA
Agona	Pig slaughterhouse	41.7 (H)	-
Anatum	Bovine	-	41.7 (H)
Enteritidis	Human	-	50.0 (H)
Give	Animal farm	63.9 (H)	-
	Dairy farm	50.0 (H)	-
Kedougou	Pig slaughterhouse	44.4 (H)	-
Kentucky	Dairy farm	38.9 (H)	-
	Chicken farm	41.7 (H)	-
Typhimurium	Pig slaughterhouse	47.2 (H)	-
	Chicken farm	55.6 (H)	-
Weltevreden	Pig slaughterhouse	69.4 (H)	-
	Chicken farm	58.3 (H)	-
Dublin	Bovine	-	19.4 (M)
Kentucky	Seafood processing plant	13.9 (M)	-
Rissen	Pig slaughterhouse	27.8 (M)	-
Virchow	Human	-	11.1 (M)
Weltevreden	Human	-	13.9 (M)
Agona	Bovine	-	No lysis
Anatum	Seafood processing plant	8.3 (L)	-
Braenderup	Human	-	No lysis
Cerro	Bovine	2.8 (L)	-
Corvalis	Animal farm	2.8 (L)	-
	Human	2.8 (L)	-
Dublin	Dairy farm	2.8 (L)	-
Heidelberg	Human	-	No lysis
Infantis	Human	-	No lysis
Javiana	Human	-	8.3 (L)
Kentucky	Bovine	-	5.6 (L)
Krefeld	Seafood processing plant	No lysis	-
Mbandaka	Dairy farm	5.6 (L)	-
	Chicken farm	No lysis	-
	Bovine	-	No lysis
Montevideo	Bovine	-	No lysis
	Bovine	-	No lysis
Muennster	Bovine	-	2.8 (L)
Newport	Bovine	-	2.8 (L)
Oranienburg	Human	-	No lysis
Ordonez	Seafood processing plant	5.6 (L)	-
Panama	Human	-	2.8 (L)
Saintpaul	Human	-	No lysis
Stanley	Swine farm	2.8 (L)	-
	Pig slaughterhouse	5.6 (L)	-
	Seafood processing plant	No lysis	-
	Human	-	No lysis
Tennessee	Seafood processing plant	2.8 (L)	-
Typhimurium	Human	-	5.6 (L)
Virchow	Human	5.6 (L)	-

^a^ Phage susceptibility level defined by % of total phages that could lyse each host strain (*n* = 36): <10% (Low; L); 11–30% (Medium; M); >31% (High; H). If no strain tested, ‘-’ is shown in table.

**Table 5:** Lysis ability and efficiency of plating (EOP) of *Salmonella* phages on *S.* Enteritidis and *S.* Typhimurium.

*Salmonella* Phage	Reference *Salmonella* Serovar	Target *Salmonella* Serovar	Lysis Ability ^a^						Level of EOP ^b^
			Phage Titer (PFU/mL)						
			10^7^	10^6^	10^5^		10^4^	10^3^	
KP1	Anatum (A4-525)	Enteritidis	+++	++	-		-	-	Medium
		Typhimurium	++	-	-		-	-	Medium
KP2	Anatum (A4-525)	Enteritidis	+++	+++	++		-	-	Medium
		Typhimurium	+++	++	-		-	-	Low
KP4	Anatum (A4-525)	Enteritidis	+++	+++	+++		+	-	Medium
		Typhimurium	+++	+++	+++		+	-	Medium
KP5	Anatum (A4-525)	Enteritidis	+++	+++	+++		+	-	Medium
		Typhimurium	+++	+++	++		-	-	Medium
KP9	Anatum (A4-525)	Enteritidis	+++	+++	+++		++	-	High
		Typhimurium	+++	+	-		-	-	Low
KP34	Virchow (H2-117)	Enteritidis	+++	+++	++		-	-	Medium
		Typhimurium	++	-	-		-	-	Low
KP36	Virchow (H2-117)	Enteritidis	+++	+++	+++		++	-	Medium
		Typhimurium	++	-	-		-	-	Low
KP49	Agona (H2-016)	Enteritidis	++	-	-		-	-	Low
		Typhimurium	++	-	-		-	-	Low
KP50	Agona (H2-016)	Enteritidis	+++	+++	+++	+++		+	High
		Typhimurium	+++	+++	+++	+		-	Medium

^a^ Clear zone or visible plaques were observed as +++, confluent lysis (clear spot); ++, semi-confluent lysis (semi-clear); +, turbidity without plaque formation. ^b^ EOP values were presented in 3 levels: high production (EOP ≥ 0.5), medium production. (0.01 ≤ EOP < 0.5) and low production (0.0001 < EOP < 0.01).

**Table 6:** Latent period and burst size of *Salmonella* phages included in a phage cocktail preparation.

*Salmonella* Phage ID	Latent Period (min)				Burst Size (PFU/Cell)			
	*S.* Enteritidis		*S.* Typhimurium		*S.* Enteritidis		*S.* Typhimurium	
	MOI 100	MOI 10	MOI 100	MOI 10	MOI 100	MOI 10	MOI 100	MOI 10
KP4	5	10	15	10	25.1	16.6	70.8	19.1
KP5	15	15	10	15	30.1	6.6	173.7	19.1
KP50	40	10	15	10	97.7	37.2	112.2	28.8

**Table 7:** Lysis ability of a phage cocktail and individual phages included in a phage cocktail preparation on *S.* Enteritidis (SE) and *S.* Typhimurium (ST) after being treated with a phage cocktail.

Treatment	Lysis Ability ^a^								
	Cocktail		KP4		KP5			KP50	
	Phage Titer (PFU/mL)								
	10^7^	10^6^	10^7^	10^6^	10^7^	10^6^	10^7^		10^6^
Control SE (non-phage treatment)	++	++	++	++	++	++	++		+
Control ST (non-phage treatment)	++	+	++	+	++	−	+		−
Phage-treated SE	++	++	++	−	++	−	++		−
Phage-treated ST	++	+	++	+	++	−	+		−

^a^ Clear zone or visible plaques were observed as +++, confluent lysis (clear spot); ++, semi-confluent lysis (semi-clear); +, turbidity without plaque formation.

**Table 8:** Estimated genome size of *Salmonella* phages isolated from animal farms in this study and *Salmonella* phage isolated from previous studies.

Source of Isolation	Country	Estimated Genome Size (kb)	Reference
Goat feces	Thailand	200 ± 2	This study
Bovine feces	Thailand	50 ± 2, 60 ± 2	This study
Poultry feed	Thailand	60 ± 2, 72 ± 2	This study
Soil (poultry farm)	Thailand	50 ± 2, 60 ± 2, 105 ± 2	This study
Drinking water (poultry farm)	Thailand	97 ± 2	This study
Poultry feces	Thailand	50 ± 2, 60 ± 2, 103 ± 2	This study
Dairy farms	USA	22 to 156	[4]
Dairy farms	Thailand	40 to 200	[31]
Sewage effluent	UK	40, 48.5 and 155	[37]
Chicken feces	Korea	240	[38]
Sewage (poultry farm)	Korea	40	[39]
Water buffalo feces	Southern Italy	39	[40]
Swine lagoon effluent (poultry farm)	UK	42	[41]
